# Altered Vitamin D Status and Bone Mineral Density in Obese and Non-obese Patients With Polycystic Ovary Syndrome: A Cross-Sectional Study in Turkey

**DOI:** 10.7759/cureus.50464

**Published:** 2023-12-13

**Authors:** Ayşen Akkurt Kocaeli

**Affiliations:** 1 Endocrinology and Metabolism, Bursa City Hospital, Bursa, TUR

**Keywords:** vitamin d deficiency, bone mineral density, vitamin d, obesity, polycystic ovary syndrome

## Abstract

Background

Polycystic ovary syndrome (PCOS) is the most common endocrinological disease in women of reproductive age. In addition to providing the basis for comorbidities such as metabolic and cardiovascular diseases, it also affects bone metabolism. This study aimed to determine whether there is a relationship between bone mineral density (BMD), vitamin D status, insulin resistance, sex hormones, and calcium metabolism disorders in women with PCOS.

Methodology

Fifty-six non-obese women with PCOS, 67 obese women with PCOS, and 45 normal weight controls participated in the study. Circulating levels of gonadotropins, estradiol, prolactin, dehydroepiandrosterone sulfate, total testosterone, thyroid stimulating hormone, sex hormone-binding globulin, insulin, glucose, and calcium metabolism parameters were assessed. We used the Homeostatic Model Assessment-Insulin Resistance Index to detect insulin resistance. BMD values in the various body regions were measured by dual X-ray absorptiometry.

Results

Women with PCOS had significantly lower vitamin D values and lumbar spine BMD than controls (p <0.001 and p <0.05, respectively). Among the patients with PCOS subgroups, vitamin D deficiency (VDD) was more frequent in obese PCOS patients (67.1%) than in non-obese patients (58.9%). We found significantly lower BMD at all sites only in the subgroup of the non-obese PCOS women than in controls (p <0.001).

Conclusions

VDD is prevalent in PCOS women in those with obesity and hyperandrogenemia. Non-obese PCOS women have significantly lower BMD measurements than healthy controls, but obese PCOS women have BMD values comparable with normal-weight eumenorrheic controls. Body mass index is the most important factor determining BMD in women with PCOS.

## Introduction

One of the most common endocrinopathies in women of reproductive age is polycystic ovary syndrome (PCOS) (5-18%), which is characterized by menstrual irregularity, polycystic ovaries, and elevated androgen levels [1]. Hormonal and metabolic changes in PCOS may also lead to vitamin D and bone metabolism disorders [2].

Vitamin D is an essential regulator of bone and mineral homeostasis. It induces calcium and phosphorus absorption in the intestines and directly affects bone formation. The risk of osteoporosis and fracture increases due to increased vitamin D deficiency (VDD)-induced parathyroid hormone (PTH). In women with PCOS, VDD is a common problem, with 67-85% of women with PCOS having 25-hydroxy vitamin D (25-OHD) values <20 ng/mL [3]. In a meta-analysis, PCOS patients have lower serum vitamin D levels than normal subjects. These variables are even lower in obese PCOS patients [3]. High expression of the vitamin D receptors in adipocytes leads to the retainment of fat-soluble vitamin D within adipose tissue, hindering its processing to a biologically active form. Observational studies revealing the association of low 25-OHD concentrations with ovulation, menstrual irregularities, and hyperandrogenism suggest that VDD may be associated with PCOS symptoms [4].

Hyperandrogenemia, high estrogen concentration, hyperinsulinemia, and a tendency to increase body mass index (BMI) help protect PCOS patients from osteoporosis, and the propensity to fractures decreases [5]. In previous studies, hyperandrogenemia, seen in approximately 75% of patients with PCOS, has been shown to positively affect bone by connecting to bone-related androgen receptors or indirectly by converting to estrogen through peripheral aromatization [6].

The positive relationship between obesity, adiposity, and BMD has been emphasized, which is frequently observed in patients with PCOS; insulin resistance (IR) and increased insulin levels reduce sex hormone-binding globulin (SHBG) and increase the amount of free androgens [7]. Insulin also reduces bone formation by increasing osteoprotegerin production from osteoblasts. This released molecule also has a resorption-increasing effect on osteoclasts. These hormones trigger osteoblast transformation and increase extracellular matrix production and mineralization [5]. Although these hormones positively impact bone individually, their effects on bone in the impaired hormonal and metabolic environment in PCOS are controversial. In addition, VDD, long-lasting amenorrhea, hypercortisolemia resulting from hyperactivation of the hypothalamic-pituitary-adrenal axis, and impaired growth hormone secretion may negatively affect bone in PCOS patients [5].

There are conflicting results in different studies in the literature. Studies show an increase [8], a decrease [9-12], or no effect [13-15] on BMD in PCOS patients compared to normal women.

In this study, we aimed to compare the BMD and 25-OHD values ​​of obese and non-obese PCOS patients with the values ​​of normal healthy women and to investigate the connection between hormonal and metabolic disorders and BMD.

## Materials and methods

We evaluated 123 consecutive premenopausal patients with PCOS and 45 age- and BMI-matched healthy controls. The study was done at the Endocrinology and Metabolism outpatient clinic, Health Sciences University Bursa City Hospital, Turkey, between March 2022 and September 2022. The study was approved by the local Ethics Committee of Bursa City Hospital (approval number 2022-18/1) and was performed per the Declaration of Helsinki. Written informed consent was collected from all subjects. According to the revised Rotterdam Criteria in 2004, participants were diagnosed with PCOS if at least two of the following three parameters were present: (1) chronic oligo/anovulation (oligomenorrhea; more than 45 days between menstruations or eight or fewer periods per year); (2) clinical presence of hyperandrogenism acne, hirsutism [modified Ferriman-Gallwey score (mFGS) ≥8 (out of 36)], androgenic alopecia, acanthosis nigricans and/or hyperandrogenism as a laboratory finding (increase in serum total testosterone (TT) levels); (3) ultrasonographic polycystic ovary image (2-9 mm diameter, 12 or more follicles, and/or increased ovarian volume (>10 mL) [16].

Exclusion criteria included (a) having adrenal or ovarian virilizing tumors, Cushing's syndrome, non-classical adrenal hyperplasia, and hyperprolactinemia findings; (b) insulin sensitivity or resistance, a disease that affects bone mineral metabolism; (c) those taking oral contraceptives, oral antidiabetic medications or vitamin D supplementations in the six months before the study; (d) use of any medication that may alter androgen levels and bone mineral metabolism within the past year; (e) smoking and consuming alcohol. 

The control group constituted participants without clinical and laboratory findings of hyperandrogenemia, BMI <25 kg/m2, and regular menstrual cycles (28 to 36 days for at least the last six months). Physical examination for hyperandrogenism by the endocrinologist evaluated all subjects. The presence or absence of masculine hair loss, excessive hair growth, and acne were recorded. Height and body weight were measured without shoes and in light clothing. BMI was calculated by dividing body weight by the square of height (kg/m2). Waist circumference (WC) was measured at the narrowest point between the iliac crest and the costal margin, and hip circumference was measured at the top of the hip while standing and breathing. Afterward, the waist-to-hip ratio (WHR) was calculated.

Participants' blood values ​​were measured in the morning following ≥12 hours of fasting, during the follicular phase of the spontaneous or progestin-induced menstrual cycle (between the second and fifth days). Homeostatic Model Assessment-Insulin Resistance Index (HOMA-IR): (fasting glucose (mg/dl) x fasting insulin (mU/l))/405 was used to measure IR for all individuals. Plasma levels of luteinizing hormone (LH), follicle-stimulating hormone (FSH), 17b-estradiol (E_2_), dehydroepiandrosterone sulfate (DHEAS), SHBG, prolactin (PRL), androstenedione, TT, PTH, thyroid stimulating hormone (TSH), 25-OHD and insulin were measured using electrochemiluminescence methods on the Cobas e411 analyzer (Roche Diagnostics Corporation, Sisli, Turkey). Total plasma alkaline phosphatase (ALP), calcium (Ca), and phosphorous (P) values were determined using calorimetric methods, and fasting glucose was measured by an enzymatic UV test-hexokinase method on the Cobas c702 (Roche Diagnostics Corporation). The 25-OHD level was classified as deficient (≤20 ng/ml), insufficient (>20 to <30 ng/ml), or sufficient (≥30 ng/ml) according to the Endocrine Society Clinical Guidelines [17]. We measured 25-OHD concentrations on serum samples of controls and patients drawn from June through early September to avoid the interference of seasonal variations of values.

BMD values in the lumbar spine (LS), femur total (FT), and femur neck (FN) sites were measured by the dual X-ray absorptiometry (DXA) method using a Hologic Delphi W (serial no: 70232; Hologic, Marlborough, MA, USA) bone densitometer. Results of Z-score and BMD are demonstrated as absolute values (g/cm2). Z-scores express the standard deviations by which a patient's value differs from a normal reference Turkish population matched for sex and age.

Statistical analysis 

We used the Statistical Package for Social Sciences (SPSS) version 23 (IBM Corp., Armonk, NY, USA) program to compare the variables. For descriptive statistics, continuous variables were expressed as mean ± standard deviation and median (minimum and maximum), and categorical variables were expressed as frequency and percentages. Categorical variables were compared with the Chi-square test. Normal distribution was tested using the Shapiro-Wilk test. The mean levels of variables with normal distribution were compared using Student's t-test or analysis of variance (ANOVA) and those without a normal distribution by Mann-Whitney U test. The Pearson (parametric) correlation tests investigated correlations between the variables. Linear regression analyses used LS-BMD, FN-BMD, and FT-BMD values as predictors. The level of significance was accepted as p<0.05.

## Results

One hundred twenty-three women with PCOS and 45 healthy women without PCOS were evaluated for the study. Sixty-seven (54.5%) out of the 123 PCOS women were obese, with a BMI ≥25 kg/m2, and 56 (45.5%) were non-obese with a BMI <25 kg/m2. All controls had regular menstrual cycles, whereas 87 (70.7%) PCOS women had oligo- or amenorrhea. Ninety-three (75.6%) of the women with PCOS had an mFGS ≥8, 51 (41.5%) had hyperandrogenemia (serum TT value >0.68 ng/ml), of which 45/67 (67.2%) were in the obese group, and 87 (70.7%) had polycystic ovaries evaluated by transvaginal ultrasound. The comparison between demographic, anthropometric, hormonal, and biochemical variables between the PCOS and control groups is demonstrated in Table 1. Demographic variables such as mean age, BMI, WC, and WHR were comparable in both groups (p>0.05). mFGS, calculated for hirsutism evaluation, was significantly higher in PCOS women than in the control group (15.31±6.07 vs. 2.57±3.39, p<0.001, respectively). The number of menstrual cycles of patients with PCOS in the last year was found to be lower than that of the control group (7.9±2.85 vs. 12.0±0.0 cycles; p<0.001). Mean HOMA-IR and insulin levels were significantly higher in PCOS women compared to controls (3.44±1 .93 vs. 1.58±1.25, p<0.001; 16.40±8.67 IU/mL vs. 8.09±5.23 IU/mL, p<0.001, respectively).

**Table 1:** Demographic, biochemical, and hormonal parameters of women between groups Data are expressed as mean ± SD*. *Statistically significant values (p<0.05) are shown in bold. PCOS: polycystic ovary syndrome; BMI: body mass index; WC: Waist circumference; WHR: Waist-to-hip ratio; mFG: modified Ferriman–Gallwey; HOMA-IR: Homeostatic Model Assessment–Insulin Resistance Index; LH: luteinizing hormone; FSH: follicle-stimulating hormone; E2: 17b-estradiol; TT: total testosterone; DHEAS: dehydroepiandrosterone sulfate; PRL: prolactin; SHBG: sex hormone-binding globulin; TSH: thyroid-stimulating hormone; Ca: calcium; P: phosphorous; ALP: alkaline phosphatase; PTH: parathyroid hormone; 25-OHD: 25-hydroxy vitamin D

	PCOS group (n = 123)	Control group (n = 45)	p-value
Demographic parameters			
Age, years	28.13±4.43	26.53±2.67	0.541
BMI, kg/m²	26.2±7.54	23.2±3.50	0.423
WC, cm	79.15±4.97	74.50±7.71	0.134
WHR	0.88±0.06	0.79±0.25	0.841
No. of menstrual cycles/year	7.9±2.85	12.0±0.0	<0.001
mFG score	15.31±6.07	2.57±3.39	<0.001
Glycemic parameters			
Fasting blood glucose, mg/dL	85.39±4.61	75.67±6.21	<0.001
Fasting blood insulin, IU/mL	16.40±8.67	8.09±5.23	<0.001
HOMA-IR	3.44±1 .93	1.58±1.25	<0.001
Gynecological hormonal parameters			-
FSH, mIU/mL	5.22±1.67	6.13±1.23	<0.001
LH, mIU/mL	9.17±5.77	5.19±2.41	<0.001
E_2_, pg/mL	45.63±18.64	87.03±13.38	<0.001
TT, ng/mL	0.75±0.18	0.26±0.09	<0.001
DHEAS, mg/dL	231.4±117.7	219.7±73.4	<0.001
Androstenedion, ng/mL	2.83±1.94	2.66±1.27	0.301
PRL, ng/mL	9.61±4.98	10.6±5.69	0.791
SHBG, nmol/L	41.78±11.27	64.51±19.46	<0.001
Ca metabolism parameters			
Ca, mg/dL	9.88±1.69	9.59±0.61	0.958
P, mg/dL	3.78±0.19	3.53±0.51	0.621
ALP, IU/L	69.45±31.15	68.51±37.1	0.788
TSH, uIU/ml	2.62±0.67	1.87±0.75	0.433
PTH, pg/ml	83.28±31.48	61.22±36.4	<0.05
25-OHD, ng/ml	13.79 ± 6.99	21.45±7.29	<0.05
<20, ng/ml, n (%)	78(63.4)	21(46.7)	<0.001
20–29.9, ng/ml, n (%)	30(24.4)	6(13.3)	<0.005
≥30, ng/ml, n (%)	15(12.2)	18(40.0)	<0.001

PCOS participants showed significantly lower mean values of FSH, E2, and SHBG in comparison to the controls (5.22±1.67 mIU/mL vs. 6.13±1.23 mIU/mL, p<0.001; 45.63±18.64 pg/mL vs. 87.03±13.38 pg/mL, p<0.001, and 41.78±11.27 nmol/L vs. 64.51±19.46 nmol/L, p<0.001, respectively). Compared with controls, women with PCOS had higher mean serum levels of LH, TT, and DHEAS (p<0.001). Both groups had similar mean plasma concentrations of TSH, androstenedione, and PRL (Table 1).

**Figure 1:**
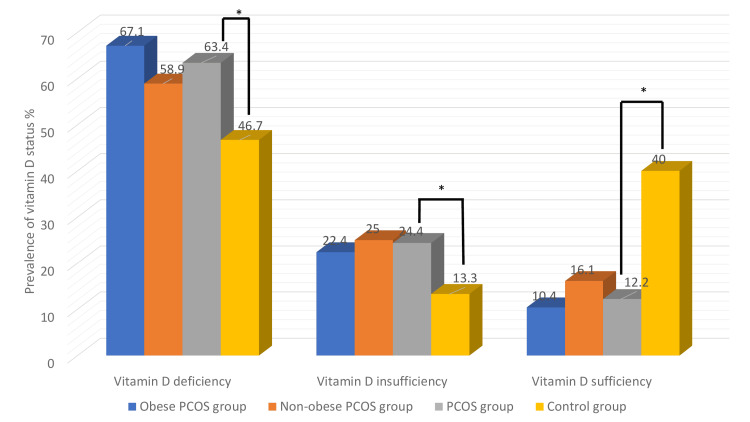
The prevalence of deficient, insufficient, and sufficient vitamin D status in women with polycystic ovary syndrome (obese and non-obese subgroups) and controls *p<0.001; PCOS vs. control group PCOS: polycystic ovary syndrome

The differences between PCOS and controls regarding several Ca metabolism parameters findings, such as ALP, Ca, and P, were insignificant (p>0.05). In contrast, the differences between the two groups regarding the PTH and 25-OHD values were significant (p<0.05) (Table 1). Women with PCOS had significantly lower vitamin D values than controls (13.79±6.99 ng/ml vs 21.45±7.29 ng/ml, p<0.001, respectively). Moreover, there was no significant difference in mean vitamin D levels between PCOS subgroups (12.4±2.80 ng/ml in obese patients with PCOS and 15.3±8.1 ng/ml in non-obese patients with PCOS, p=0.72). Vitamin D insufficiency and deficiency were more frequent in patients with PCOS than in controls (24.4% vs. 13.3%, p<0.005; 63.4% vs. 46.7%, p<0.001, respectively). Additionally, the prevalence of adequate 25-OHD concentrations in PCOS women was significantly lower than in controls (12.2% vs. 40%, p<0.001, respectively). Among the patients with PCOS subgroups, vitamin D deficiency was more frequent in obese PCOS patients (45 out of 67, 67.1%) than in non-obese patients (33 out of 56, 58.9%), although this was not statistically significant (p>0.05). The prevalence of deficient, insufficient, and sufficient prevalence of 25-0HD status in PCOS patients (obese and non-obese subgroups) and controls are shown in Figure 1.

In the correlation analysis, serum 25-OHD level was significantly negatively correlated with BMI (r=-0.243, p=0.04), WHR (r=-0.213, p=0.023), HOMA-IR (r=-0.537, p=0.002), PTH (r=-0.118, p=0.023), DHEAS (r=-0.554, p=0.023), and mFG scores (r=-2.95, p=0.033). There was a significant positive correlation between plasma 25-OHD level and SHBG (r=1.95, p=0.024). When women with PCOS were grouped as hyperandrogenemia (51/123, 41.5%) or non-hyperandrogenemia (72/123, 58.5%), 25-OHD values were significantly lower in women with hyperandrogenemia than in those without hyperandrogenemia (10.79±5.32 vs. 16.49±3.65 ng/ml, p<0.01 respectively).

The BMD measurement results are summarized in Table 2. The BMD and Z-score values did not differ between the patients with PCOS and control subjects at any of the sites except for LS-BMD, which was lower than controls (1.15±0.42 vs. 1.45±0.07 g/cm2, p<0.01, respectively). Additionally, our study analyzed whether changes in body weight affected BMD. LS, FN, and FT-BMD values were determined to be significantly higher in obese patients with PCOS than in non-obese patients with PCOS (Table 2). Furthermore, BMD measurements ​​of non-obese women with PCOS were significantly lower than normal weight controls at all sites (Table 2). However, BMD and Z-score values ​​of obese individuals with PCOS were comparable to the controls in all regions (p>0.05). 

**Table 2:** Absolute bone mineral density and Z-scores of various body regions of women between groups Data are expressed as mean ± SD. *p<0.01 compared to non-obese PCOS patients; ^†^p<0.01 and ^††^p<0.001 compared to controls; ^§^p<0.05 compared to controls BMD: bone mineral density; PCOS: polycystic ovary syndrome

	Obese PCOS group (n=67)	Non-obese PCOS group (n = 56)	PCOS group (n = 123)	Control group (n = 45)
BMD (g/cm^2^)				
Lumbar spine	1.26±0.55*	0.97±0.23^†^	1.15±0.42^§^	1.45±0.07
Femur neck	0.92±0.01*	0.79±0.33^†^	0.84±0.13	1.07±0.26
Femur total	1.09±0.04*	0.82±0.35^††^	1.01±0.22	1.29±0.31
Z-score				
Lumbar spine	-0.04±1.27	-0.59±1.78	-0.24±1.27	0.15±0.42
Femur neck	0.06±1.18	-0.13±1.09	0.07±0.98	0.19±1.20
Femur total	0.13±0.41	-0.05±1.13	0.06±0.41	0.21±1.12

In the PCOS group, LS, FN, and FT-BMD were positively correlated with BMI (r=0.325, p<0.01; r=0.765, p<0.01 and r=0.124, p<0.001, respectively). Additionally, HOMA-IR and LS-BMD had a significant positive correlation (r=0.215, p<0.01). This study found no significant correlation between the Ca metabolism parameters and the BMD values of LS, FN, and FT (p>0.05).

In women with PCOS, LS-BMD was significantly positively correlated with serum E2 (r=0.321, p<0.01) and TT (r=0.236, p<0.01) levels. No significant correlation was found between the BMD of all regions and DHEA-S and SHBG values.

## Discussion

This study aimed to demonstrate the correlation of altered BMD and vitamin D status with metabolic changes, sex steroids, and androgens in obese and non-obese PCOS patients compared to healthy women. 

Vitamin D is well known for maintaining Ca and P homeostasis and promoting bone mineralization. In addition, the presence of vitamin D receptors in the endometrium and ovary suggests that they also play a role in steroidogenesis in the reproductive system [18]. In this study, consistent with some studies [19,20], serum 25-OHD levels were significantly lower, and the prevalence of 25-OHD insufficiency and deficiency was statistically significantly higher in patients with PCOS compared to control subjects. There are conflicting results in studies comparing vitamin D levels in patients with and without PCOS. A cross-sectional study was conducted in Shaanxi, China, including 169 patients diagnosed with PCOS and 114 healthy participants [21]. Serum 25-OHD level was significantly lower in women with PCOS than in controls (11.6±7.2 vs. 18.9±8.4 ng/mL, p<0.05) [21]. Lejman-Larysz et al. [22] found the mean vitamin D levels of PCOS patients to be similar to the control group. Differences between studies can be explained by the different criteria for diagnosing PCOS, the differences between the geographical regions where the studies were conducted, and the more regular use of vitamin D supplements in developed countries.

In studies, VDD has been found more frequently in obese people, and a negative correlation between the 25-OHD levels and BMI has been shown in patients with PCOS by most authors [19,23]. Since obese individuals spend less time outdoors, their exposure to sunlight decreases, and the conversion of cholecalciferol, the precursor of vitamin D, from cholesterol, reduces. In addition, the availability of 25-OHD, a fat-soluble vitamin, decreases in proportion to the increase in adipose tissues [3]. Velija-Asimi [23] found 25-OHD <20mg/dl in 68% of patients with PCOS (41 out of 60 patients) and emphasized that more than half of these patients were obese. In our study, consistent with literature data, although there was no statistically significant difference between mean vitamin D values in PCOS subgroups, VDD was found more frequently in obese PCOS patients than non-obese patients.

In the correlation analysis performed in our study, a significant negative correlation was shown between vitamin D values and BMI, HOMA-IR, WHR, and PTH. There are conflicting results about the relationship between VDD and IR. Differences between studies may result from a lack of a more homogeneous distribution of populations or technical deficiencies.

Hyperandrogenism is one of the main symptoms in patients with PCOS, and serum TT, DHEAS, SHBG levels, and free androgen index (FAI) are used for diagnosis. Studies show a positive or negative relationship between low serum vitamin D concentrations and hyperandrogenism markers [3]. A positive correlation between plasma vitamin D levels and SHBG has been demonstrated in PCOS patients [20], and a negative correlation with TT, DHEAS, and FAI levels [24]. In a recent study, among PCOS women with VDD, females aged <26 and overweight had higher risks of hyperandrogenemia [25]. In our study, vitamin D levels were significantly lower in women in the hyperandrogenemia group than those without. More than half of the patients with hyperandrogenemia were obese (67.2%), which may also explain the low vitamin D level.

We also evaluated the BMD of the patients in terms of the risk of osteoporosis. It was observed that only the lumbar spine BMD of individuals with PCOS was statistically significantly lower than the healthy controls. In this study, the PCOS patients were divided into subgroups according to their BMI to evaluate the impact of the BMI on the BMD. Although the BMD values ​​of non-obese women with PCOS were significantly lower than those of normal weight controls at all regions, the BMD and Z-scores of obese individuals with PCOS were similar to controls. Additionally, LS, FN, and FT-BMD values were measured to be significantly higher in obese patients with PCOS than in non-obese patients with PCOS. BMI is an essential factor affecting BMD in patients with PCOS. Increased biomechanical forces on bone and increased aromatization of androgens in adipose tissue explain the positive relationship between body weight and BMD.

Our results support the findings of To et al. [10], Katulsky et al. [11], and Karadağ et al. [12], who also found that patients with PCOS had lower BMD values than controls. Contrary to many previous studies, there are also studies showing that BMD is similar between women with PCOS and controls [13-15]. These contradictory results between studies may be due to different PCOS diagnostic criteria, as well as different inclusion criteria for women with PCOS. For example, patients' mean age, BMI, and menstrual cycles may vary. 

Some studies have investigated whether weight has any effect on BMD in normal-weight or obese PCOS patients [10,15]. Parallel to our results, in a study conducted by Ganie et al. [14] in which 60 PCOS patients were evaluated, FN and lumbar vertebra BMD values ​​of obese PCOS cases were found to be significantly higher than those of overweight and normal-weight PCOS cases. A positive correlation between BMI and BMD was also shown. In a recent meta-analysis of 21 studies with PCOS, women with BMI <27 kg/m^2^ had lower FT and LT-BMD measurements than the control group. On the other hand, the BMD value of women with PCOS with BMI >27 kg/m^2^ was comparable with the control group [26]. In the study conducted by Kalutski et al. [11], which included 69 PCOS patients, it was concluded that, unlike the results of our research, the BMD values ​​of overweight/obese PCOS cases (n=17) did not differ from those of normal-weight PCOS cases. The lack of difference can be explained by the relatively low number of obese patients.

Studies have revealed that IR in PCOS patients positively affects BMD [9,12]. Insulin is an anabolic hormone for bone, and its excess affects bone metabolism in patients with PCOS. Insulin has a direct effect on osteoblasts and osteoclasts. It also increases the impact on BMD by reducing SHBG and increasing free estrogen and testosterone levels. In addition, it increases the sensitivity of osteoblasts to insulin-like growth factor-1 (IGF-1) by suppressing IGF-binding peptide-1 (IGFBP-1) [27]. We also found that HOMA-IR and LS-BMD had a significant positive correlation in patients with PCOS, in line with previous studies.

A positive effect of androgens on BMD has been demonstrated through various mechanisms. The effects of androgens on bone occurs through receptors or by aromatization in peripheral fat tissue. They also reduce interleukin-6 (IL-6) production, inhibit prostaglandin production, and suppress the effect of PTH on osteoblasts [28]. We have found a positive correlation between TT concentrations and BMD in PCOS participants. Our results agree with the results of Noyan et al. [13]. However, Katulsky et al. [11] and Ganie et al. [14] demonstrated no correlation between BMD and serum TT levels. These contradictory results between TT and BMD levels may be because testosterone analyses are performed with different methods, and BMI, HOMA-IR, PTH, and 25-OHD values, which may affect BMD values ​​in PCOS patients, are not distributed homogeneously in the studies [29].

Some limitations should be considered when interpreting this study’s result because our research was conducted in a single clinic, and the population was monoethnic (i.e., Turkish Caucasian). Therefore, our results may not apply to other populations with PCOS. The impact of ethnic differences, especially on the presence or severity of hirsutism, cannot be ignored. Secondly, the mean age of the individuals in our study was <35 years, and peak bone mass had not yet been reached. Thirdly, bone turnover markers were not measured in our study. Another limitation was that the sample size was relatively small. Further long-term and multicenter studies with larger sample sizes are needed to investigate the causal relationship between vitamin D, BMD, and hyperandrogenism. 

## Conclusions

We have shown significantly lower vitamin D and LS-BMD values in patients with PCOS compared to control women. Obese PCOS women had significantly higher BMD compared to non-obese women. While there was a positive correlation between IR, BMI, E2, TT, and BMD, no correlation was detected between BMD and Ca metabolism parameters, DHEA-S, and SHBG values. We think that in PCOS cases where estrogen and androgens are at unbalanced levels and accompanied by metabolic problems such as insulin resistance, the mechanisms by which these factors affect bones have not been fully elucidated. We believe that prospective, clinical, and molecular multicentric studies will be useful for this purpose.
